# Thermal property evaluation of a 2.5D integration method with device level microchannel direct cooling for a high-power GaN HEMT device

**DOI:** 10.1038/s41378-022-00462-3

**Published:** 2022-11-11

**Authors:** Tingting Lian, Yanming Xia, Zhizheng Wang, Xiaofeng Yang, Zhiwei Fu, Xin Kong, Shuxun Lin, Shenglin Ma

**Affiliations:** 1 Department of Mechanical and Electrical Engineering, Xiamen University, Xiamen, 361005 China; 2 China Electronic Product Reliability and Environmental Testing Research Institute, Guangzhou, 510000 China; 3 The 29th Research Institute of China Electronics Technology Group Corporation, Chengdu, 610000 China; 4 Chengdu HiWafer Semiconductor Co., Ltd., Chengdu, 610000 China

**Keywords:** Engineering, Electrical and electronic engineering

## Abstract

Gallium nitride high electron mobility transistor (GaN HEMT) devices have become critical components in the manufacturing of high-performance radio frequency (RF) or power electronic modules due to their superior characteristics, such as high electron saturation speeds and high power densities. However, the high heat characteristics of GaN HEMTs make device level cooling a critical problem to solve since performance degradation or even failure may occur under high temperatures. In this paper, we proposed a 2.5D integration method with device-level microchannel direct cooling for a high-power GaN HEMT device. To demonstrate this technological concept, a multigate GaN HEMT device featuring a gate length/width/source drain spacing of 0.5 μm/300 μm/6 μm that underwent in-house backside thinning and metallization was used as the test vehicle. A high-resistivity silicon (HR Si) interposer embedded with four-layer microchannels was designed, having widths/pitches of 30 μm/30 μm at the top microchannel. The high-power GaN HEMT device was soldered on a Si interposer embedded with open microchannels for heat dissipation. A pair of GSG Pad chips was soldered simultaneously to display the capacity for the heterogeneous integration of other chip types. Thermal property evaluation was conducted with experiments and simulations. The test results showed that the maximum surface temperature of the GaN HEMT device decreased to 93.8 °C when it experienced a heat dissipation density of 32 kW/cm^2^ in the gate finger area and an average heat dissipation density of 5 kW/cm^2^ was found in the active area with the DI water coolant at a flow rate of 3 mL/min. To our knowledge, among recently reported works, this finding was the best cooling capacity of heterogeneously integrated microchannels for GaN HEMT devices. In addition, this technology was scalable regarding the numbers of gate fingers or GaN HEMT devices.

## Graphical abstract

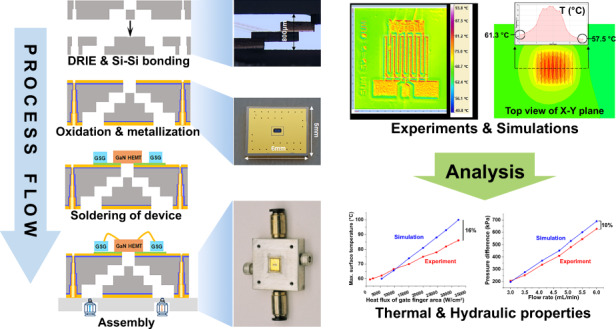

## Introduction

GaN semiconductors are widely used as substrates in radio frequency and power electronic devices, such as HEMT devices; they feature high electron saturation speeds, breakdown field strengths, power densities, and thermal conductivities. Traditionally, high-power GaN HEMT devices are mounted on high thermal conductivity carriers, such as molybdenum copper carriers^1^ and diamond carriers^2–5^, and they are further loaded on cold plates for heat dissipation. As GaN HEMT devices continue to evolve, the heat flux behind the gate finger can reach tens of kW/cm^2^, and the average heat flux can exceed 1000 W/cm^2^; therefore, the heat dissipation becomes a key challenge. If the heat is not dissipated in time, GaN HEMT devices quickly degrade or even fail^6–13^. Therefore, the thermal resistance between the GaN device and the cold plate must be reduced to keep the junction temperature within an acceptable range. A heat dissipator is proposed to be transferred inward the package or module from the outside for reducing the thermal resistance between the junction zone and the environment.

While the location of the highest joule heating in the GaN HEMT device is near the top surface, the heat dissipator on the top surface of the device is considered a potential solution. Some top-side passive cooling technologies are proposed, such as AlN passivation^14^, graphene quilts^15^, and electroformed low stress metal heat sinks^16^. Although these cooling solutions are shown to be effective, they impact the performance levels of devices and lead to difficulty in electrical interconnection. In addition, top-side active cooling (i.e., top-side microchannel cooling) can exacerbate previous problems. Another solution is double-side heat dissipation, which is very likely to exhibit better cooling performance^17^. However, this technology has low process feasibility, while the impact on the device performance and the difficulty in the electrical interconnection increases.

With little influence on the electrical performance levels of devices and relatively high feasibility, bottom-side active cooling with embedded microchannels has attracted great interest from researchers. In recent years, various kinds of bottom-side microchannel heat sinks are studied. In 2006, researchers at the US Naval Laboratory studied the cooling performance of parallel straight microchannel heat sinks fabricated on Si, AlN, SiC, and copper substrates^18^, and the best test result occurs from the SiC-based microchannel. A 1.2 × 5 mm^2^ heater is directly fabricated on the surface of a SiC-based microchannel to characterize the cooling capacity, showing a maximum surface temperature of 120 °C as the power density increases to 3000–4000 W/cm^2^. This work demonstrates the cooling capacity of microchannels; however, there is no further information about methods to integrate the microchannel with GaN HEMT devices or module assemblies at the publication time. In 2015, the Raytheon research team has proposed a conceptual GaN HEMT device on a composite substrate with microchannels inside^19–22^. The device is composed of a SiC substrate of a GaN HEMT device and a diamond or Si substrate bonded together with solder. Simulation results show that the peak temperature is controlled in the range of 182–212 °C when the GaN HEMT device experiences a heat flux of approximately 31–38 kW/cm^2^ in the gate finger zone and an average heat flux of 1 to 1.25 kW/cm^2^ throughout the device zone. However, there is no sample or test result proposed in the publication time. In 2016, researchers at Lockheed Martin proposed a method of mounting a GaN HEMT device on a manifold microchannel heat sink^1,23–25^, where the whole SiC substrate is thinned to ~100 μm and the region beneath the active area is thinned to 30 μm. When it works, the coolant is pumped through the manifold heat sink and jetted directly to the locally thinned SiC substrate. This work shows a maximum temperature increase below 120 °C when it experiences a heat flux of 30 kW/cm^2^ in the gate finger zone and an average heat flux of 1 kW/cm^2^ throughout the active area. However, the increase in the reliability risk due to the coolant directly jetting on the very thin active zone is notable. In 2018, our team demonstrated a 2.5D integrated 2–6 GHz GaN HEMT device-based power amplifier on an HR Si interposer embedded with microchannels^26^. The test results showed that the device worked normally in the range of 2–6 GHz when it experienced a heat dissipation density of 400 W/cm^2^. In 2020, researchers at EPFL proposed an approach of codesigning an electronic device with integrated microchannels on a GaN-on-Si substrate^27–29^. A full wave bridge AC-DC convertor based on Schottky diodes on a GaN layer with a cooling manifold microchannel embedded inside the Si substrate is demonstrated, showing a temperature increase of approximately 60 K when it experiences a heat flux of 1.7 kW/cm^2^ and an average heat flux of 417 W/cm^2^ at the device level upon working.

By reviewing the literature, we see that the cooling capacity of various types of microchannel heat sinks have been studied and acknowledged as potential solutions for cooling GaN HEMT devices. However, there is no authoritative conclusion regarding which of them is the best, as the enhancement of microfluidics cooling performance usually conflicts with the compatibility of the process. To fully utilize the cooling capacity of microchannels, it is natural and effective to shorten the distance between the heat source zone and the microchannel. However, this action will lead to compatibility issues and increase the reliability risks or difficulty in process development. In fact, only a few research groups can manufacture practical samples of heterogeneously integrated microchannels for GaN HEMT devices. By considering the enhanced difficulty of integrating with GaN HEMT devices, manifold microchannels featuring complicated structures might not be an ideal solution for device-level in situ cooling but are more suitable for the cooling of large-region heat sources. It is better to use a traditional parallel straight microchannel for device level cooling, which can be more easily integrated with GaN HEMT devices due to its simple structure.

From the perspective of cooling integration, the on-substrate monolithic integration of GaN HEMT devices with microchannels is the most straightforward and compact solution^30^. However, limited room and the requirement of heterogeneous integration with other chips can make packaging or assembly a very challenging issue. The situation is the opposite for traditional 2D integration (e.g., MCM, SiP), where chips and devices are directly bonded to a ceramic substrate or PCB^31^. For traditional systems where GaN HEMT devices are not used, a very feasible cooling scheme for 2D integration involves adding an additional microchannel heat sink to the system. However, in this scenario, the distance between the heat source and microchannel (i.e., heat transfer path) inevitably increases. When a GaN HEMT device, which can generate a very high heat flux of over 1 kW/cm^2^ at the device level and tens of kW/cm^2^ at the gate finger area, is used in the system, the long path of heat transfer does not allow the heat to be removed in time and finally leads to degradation or failure of the device. In addition, the size of the whole system increases considerably if an additional heat sink is added. The TSV interposer-based 2.5D integration acts as a compromise between monolithic integration and 2D integration; it has the advantages of relatively high process feasibility and integration compactness. This technology refers to bonding chips, devices, etc. to a TSV interposer and further bonding the interposer to a substrate^31,32^. By forming microchannels inside the interposer, the heterogeneous integration of various devices with embedded microchannel cooling is realized with an acceptable process difficulty.

Hence, we propose a 2.5D integration method with device-level microchannel direct cooling for a high-power GaN HEMT device in this study. To demonstrate the concept, a multigate GaN HEMT device featuring a gate length/width/source drain spacing of 0.5 μm/300 μm/6 μm is selected as the test vehicle, which is thinned on the backside, metalized and further soldered on a corresponding HR Si interposer embedded with 4-layer stepped microchannels to realize direct cooling. Key processes are identified and developed. The thermal properties of the GaN HEMT device are studied in combination with experiments and simulations to show the cooling capacity.

## Design

**Fig. 1:**
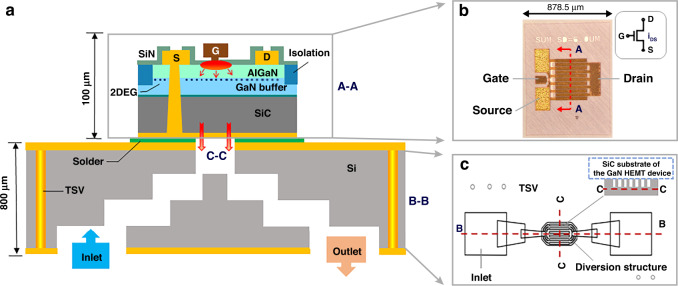
A 2.5D integration method with chip-scale integrated microchannel direct cooling for a high power GaN HEMT device. **a** Schematic illustration of the integration structure. **b** Top-side view of the GaN HEMT device and its electrical schematic diagram. **c** Schematic illustration of the HR Si interposer embedded with microchannels

According to the cooling requirements of GaN HEMT devices, a setup to realize heat dissipation is shown in Fig. 1a. A multigate GaN HEMT device fabricated on the GaN layer on a SiC substrate is selected, as shown in Fig. 1b, which has 10 gate fingers that are each 300 μm in width and 0.5 μm in length, and the whole chip size is 1120 μm (height) × 878.5 μm (width). An incoming GaN HEMT device wafer is temporarily bonded to a carrier wafer, thinned on the backside to 100 μm in thickness, and then finished sequentially via formation and backside metallization. Finally, dicing and debonding are finished. By considering the small size of the GaN HEMT device, a 4-layer stepped microchannel is designed inside an HR Si interposer, as shown in Fig. 1c. In this study, the HR Si interposer is not only designed to provide a microchannel to cool the GaN HEMT device but also works as an electrical interconnection substrate for other discrete devices or ICs because high-resistivity silicon is helpful for reducing high frequency loss. While the gate fingers of the GaN HEMT device are arranged in parallel, the top layer of the microchannel is designed as a parallel straight structure with a width/spacing of 30 μm/30 μm in a similar pattern. The gate fingers are aligned in parallel, and a direct contact is obtained between them to achieve a closer distance from the heat source zone and to improve the bonding strength. To make the coolant velocity more uniform in the parallel microchannels, a diversion structure is designed at the inlet and outlet regions.

From the top layer of the microchannel, as stated previously, the 4-layer stepped microchannels expand from 380 μm × 760 μm (top) to 700 μm × 900 μm (bottom). Rather than a sudden jump in size, the sizes of microchannels change gradually, thus the pressure drop of the system decreases. The HR Si interposer is metalized with Cu/Au on both sides, and the total thickness after metallization is approximately 800 μm. A TSV interconnection is designed for the GaN HEMT device’s electrical grounding or any further electrical interconnections with other chips to achieve heterogeneous integration. TSV is metalized with copper plating, followed by a NiPaAu coating.

## Materials and methods

### Fabrication

**Fig. 2:**
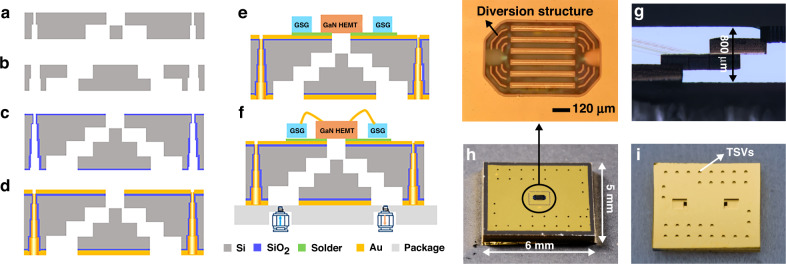
Process of HR Si interposer embedded with an opened microchannel. **a**, **b** Microchannels and TSVs formed by the DRIE process. **c** Oxidation. **d** Metallization. **e** Soldering of GaN HEMT devices and GSG Pad chips on an HR Si interposer. **f** Assembly and gold wire bonding. **g** Cross-section of HR Si interposer embedded with 4-layer microchannels. **h** Topdown photo of HR Si interposer with magnifications of parallel straight microchannels. **i** Bottom side of the HR Si interposer with inlet and oulet ports

We developed a microchannel-embedded HR Si interposer process, as shown in Fig. 2a–d. First, double-side polished silicon wafers of 400 μm were used, and they were etched with a deep reactive ion etching process to 200 μm in depth to form microchannels and TSVs on both sides sequentially. To reduce the process difficulty for TSV interconnection, vias with sequentially stepped diameters were designed, as shown in Fig. 2a, b. Second, the silicon wafers were aligned and bonded with a silicon–silicon direct bonding process to form 4-layer stepped microchannels and TSVs.

Figure 2g shows the optical micrograph of the cross-section after silicon–silicon bonding, which displayed a good bonding interface. Then, a high-temperature thermal oxidation process was performed to grow a continuous layer of SiO_2_ on the whole outside surface as an insulating layer, as shown in Fig. 2c. Finally, a double-sided sputtering process was used to form a Ti/Cu layer with a thickness of 200 nm/500 nm for metallization, followed by a Au plating process, as shown in Fig. 2d. Figure 2h shows a microscopic photo of the top side, where the microchannels and coolant diversion structures in the inlet and outlet are seen. Figure 2i shows a photo of the bottom side, where a pair of large coolant ports is seen. According to Fig. 2h, i, TSVs were shown to make both sides electrically interconnected.

The GaN HEMT device was aligned and mounted to the corresponding opened microchannels of the HR Si interposer by performing AuSn eutectic bonding, as shown in Fig. 2e. In this study, to guarantee the bonding quality with a low thermal resistance, the process was already optimized by performing X-ray tests, warpage tests, and shear tests to process samples (details can be seen in the supplemental information). A pair of GSG Pads were aligned and soldered to both sides of the GaN HEMT device simultaneously. Then, the HR Si interposer was assembled into a customized aluminum alloy box, and the gaps around the inlet and outlet regions were sealed by DOWSIL™ 3145 adhesive. Gold wire bonding was used to interconnect GaN HEMT devices and GSG Pads, as shown in Fig. 2f. In this study, only one GaN HEMT device and one pair of GSG Pad chips were assembled to verify the concept; however, more chips, such as impedance matching devices, could be assembled in the same manner to realize heterogeneous integration simultaneously.

### Experimental setup

**Fig. 3:**
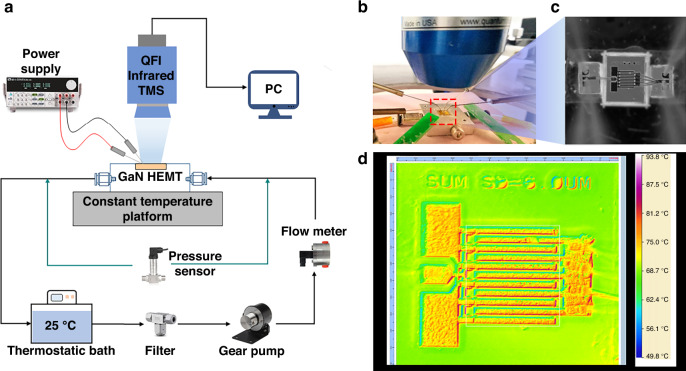
The thermal properties evaluation experiments. **a** Schematic overview of the experimental setup. **b** Close-up picture of the sample placed on the platform. **c** Magnified photo of a GaN HEMT device on the HR Si interposer. **d** Captured IR image of the GaN HEMT device during the experiment

Figure 3a shows the experimental setup used to evaluate the thermal properties of device-level integrated microchannel cooling technology for high-power GaN HEMT devices. The sample is placed on a temperature controllable platform with a pair of DC probes configured to touch the GSG pads for signal access; additionally, a QFI infrared (IR) thermal imager is configured above to capture the surface temperature image. Deionized water is chosen as the coolant, which has the advantages of high specific heat capacity and easy preparation. From a thermostatic bath, DI water is pumped by a gear pump (MICROPUMP GAF-T23) into the microchannel as coolant. DI water directly contacts the backside of the thinned GaN HEMT device and finally flows back to the same thermostatic bath. The temperature and pressure characteristics of the DI water at the inlet and outlet regions are detected by a thermocouple and a pressure sensor (GE Druck PMP5023), respectively.

To ensure accurate IR measurement and to avoid damage to the GaN HEMT device caused by coating, pixel-by-pixel emissivity correction was used^33^. Calibration was conducted to realize the emissivity correction before the experiment. The sample was heated to various temperatures by the temperature controllable platform, and IR measurement was performed by the thermal imager to acquire reference data simultaneously. Patented algorithms embedded in QFI software fit the temperature with IR emission; thus, automatic postprocessing of IR data are conducted to generate correct thermal images during the experiment. In such scenarios, IR thermography images with high accuracies are realized without coating.

In the experiment, the bottom surface of the sample is maintained at 60 °C by the temperature controllable platform to simulate the background heat in practical application scenes (e.g., mounted inside a drone). The GaN HEMT device is adjusted to work in DC mode, and the flow rate of DI water is set to 3 mL/min. The gate-to-source voltage *V*_gs_ is adjusted to 1 V to keep the GaN HEMT device fully turned on. The drain-to-source voltage *V*_ds_ increases slowly until a stable drain current *I*_ds,_ and the IR image of the device active region is monitored simultaneously. The values of *V*_ds_, *I*_ds_ and IR images are recorded accordingly. The input power *W* is calculated by *W* = *V*_ds_ *I*_ds_, and the heat flux *Q* in the gate finger area is obtained by *Q* = *W / A*. When considering the heat flux in the gate finger zone, *A* represents the gate finger area of the GaN HEMT device and is equal to the total gate width multiplied by the source drain spacing, which is 10 × 300 × 6 μm^2^. When considering the heat flux throughout the active zone, *A* represents the active area of the GaN HEMT device and is equal to 303 μm × 393 μm. Figure 3b shows the integrated microchannel-cooled GaN HEMT device under measurement, and Fig. 3c is a magnified view. Figure 3d is a captured IR image that shows a maximum surface temperature of 93.8 °C during the experiment. Finally, *V*_ds_ increases to ~7.5 V and *I*_ds_ reaches 0.8 A, yielding a heat flux of 32,806 W/cm^2^ in the gate finger zone and an average heat flux of 5044 W/cm^2^ throughout the active zone.

### Simulation

**Fig. 4:**
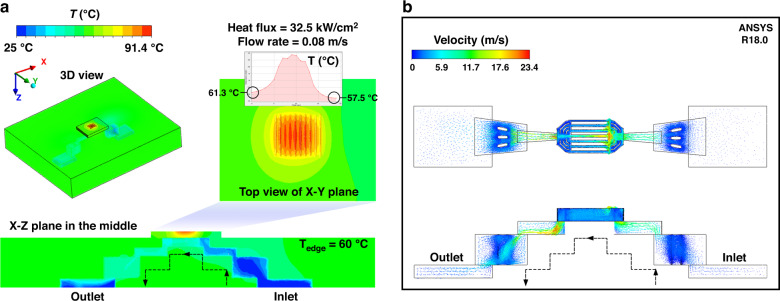
Simulation results when the heat flux on the gate finger is 32.5 kW/cm^2^ and the inflow velocity of DI water is 0.08 m/s. **a** Temperature contour of a high-power GaN HEMT device on an HR Si interposer embedded with a microchannel. **b** Velocity vector in the microchannel

To better understand the experiment, a simulation was conducted with ANSYS Workbench. In this study, the model was assembled in the same configuration as shown in Fig. 2e, in which the GSG pad was removed for simplification. The boundary conditions in the simulation were set according to the experiment. The ambient temperature was set to 27 °C, and the convective heat transfer coefficient of free air was set to 6.0 W/(m^2^K), an empirical value. The initial temperature of the system was set to 27 °C, the temperature of the bottom contact surface was set to 60 °C, the temperature of the incoming water was set to 27 °C, and the inflow velocity was set to 0.08 m/s at the start of each microchannel (equivalent to 3 mL/min in the experiment). The thermal conductivities of the materials are shown in Table 1, where *T* is the temperature of the material. By assuming that the heat flux on the gate finger was uniform, the heat flux was set to 32.5 kW/cm^2^. Figure 4a shows the temperature at the center of the gate finger of the GaN HEMT device, showing a maximum temperature of 91.4 °C. Figure 4b shows the corresponding velocity vector contour in the 4-layer microchannels. The largest flow rate was at the entrance of top parallel straight microchannels, where the corresponding pressure was 198 kPa. Compared to the measurements, the maximum temperature had an error of 16%, and the pressure difference error between the inlet and outlet was ~10%.

**Table 1:** Thermal conductivities of various materials used in simulation^1^

Materials	Thickness (μm)	Thermal conductivity (W/mK)
GaN	1	−0.1623 T + 214.17, T (in K)
SiC	99	0.0038 T^2^ - 4.1734 T + 1259, T (in K) < 600 K
Si	800	148

## Results and discussion

**Fig. 5:**
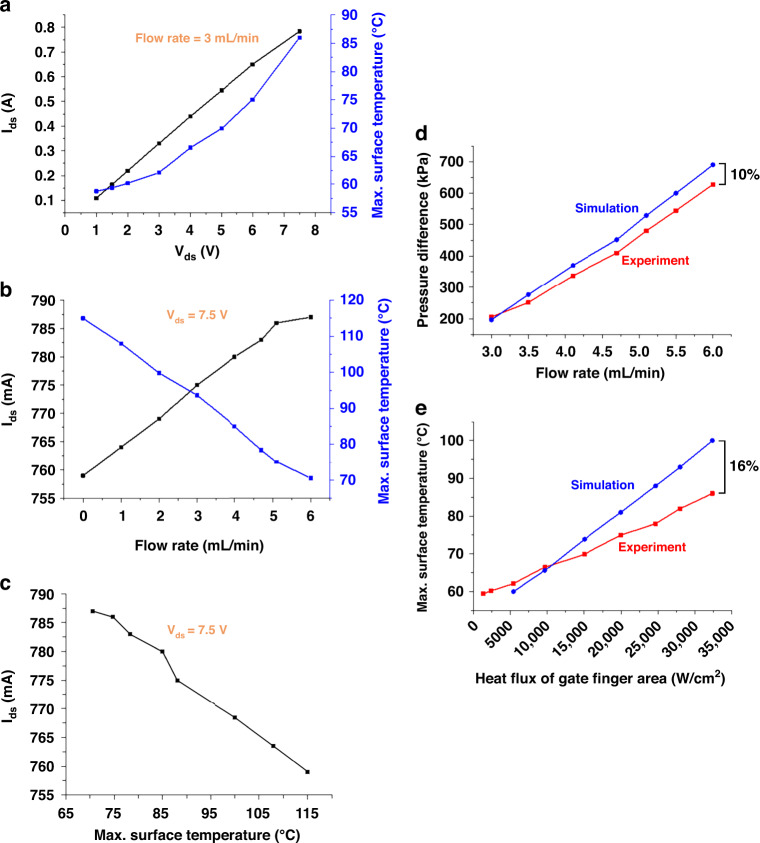
Analysis of thermal, electrical, and hydraulic properties in DC mode. **a** The drain-to-source current *I*_ds_ and the maximum surface temperature versus the drain-to-source voltage *V*_ds_ when the DI water flow rate is set to 3 mL/min. **b** *I*_ds_ and the maximum surface temperature versus DI water flow rate when *V*_ds_ is set to 7.5 V. **c** *I*_ds_ versus maximum surface temperature when *V*_ds_ is set to 7.5. **d** Pressure difference between the inlet and outlet from the simulation and the measurements. **e** The maximum surface temperature versus heat flux at the gate finger from the simulation and the measurements

*V*_ds_ and DI water flow rate are taken as variants to evaluate the thermal property of the embedded microchannel. Figure 5a, b summarizes the response of *I*_ds_ and maximum temperature to *V*_ds_ and coolant flow rate. We find that when the flow rate is fixed at 3 mL/min, as *V*_ds_ increases, *I*_ds_ linearly increases, and the maximum surface temperature increases accordingly. At a given gate-to-source voltage (*V*_gs_ = 1 V) and drain-to-source voltage (*V*_ds_ = 7.5 V), when the flow rate increases, the maximum surface temperature decreases by ~45 °C, and *I*_ds_ increases by ~30 mA, as shown in Fig. 5b. A linear relationship between *I*_ds_ and the maximum surface temperature is further deduced. As shown in Fig. 5c, when the temperature increases from 70 to 115 °C (referring to flow rates of 6 and 0 mL/min, respectively), each degree Celsius increase contributes to a decrease in *I*_ds_ of 0.67 mA. This response of current to temperature is on the same order of magnitude as the result from published work, where the decrease in *I*_ds_ is due to the increase in the carrier collision and the decrease in the mobility when the temperature increases^34^. Furthermore, a decrease in the power *W* from 5.91 to 5.69 W is found. While the performance degradation of the device at high temperatures is implied, microchannel cooling can benefit the GaN HEMT device by effectively decreasing the operating temperature, preventing degradation, and increasing the power capacity.

The measured pressure difference between the inlet and outlet regions is compared to the simulation, as shown in Fig. 5d, where the error is within 10%. At a given flow rate of 3 mL/min, the relationships between the maximum temperature and heat flux in the gate finger area are determined, as shown in Fig. 5e. The slope of simulation is generally greater than that of measurement. As the heat flux increases, two curves intersect at approximately 10 kW/cm^2^. The main reason why such a difference occurs is ascribed to the platform, which is not factored in our simulation. When the heat flux is low, the platform behaves like a heat source as it is set to 60 °C. When the heat flux is at a high level, it behaves more like a heat sink to make the simulated maximum temperature lower than that extracted from the measurement.

Table 2 compares the results in published papers. The cooling coefficient of performance (COP) in this study is defined as the ratio of power dissipation to pumping power (COP = *W/P*_pump_), where pumping power is calculated by the flow rate and pressure drop (*P*_pump_ = *f p*)^18,27^. When the flow rate is 3 mL/min and the heat flux is 32,806 W/cm^2^, the calculated best COP is 3725. When the heat flux at the gate fingers increases to 32 kW/cm^2^ in our work, the average heat flux in the whole active area is approximately 5 kW/cm^2^, and the temperature increase is only 68.8 °C. Although there is still room for improvement, this finding is already sufficient for verifying the cooling capacity of the proposed technology. In addition, since the parallel straight microchannels are aligned parallel with the gate fingers, the chip-scale integrated microchannel cooling technology are scaled up by increasing the number of gate fingers and the corresponding straight microchannels simultaneously.

**Table 2:** Comparison of the cooling capacity of microchannels published in recent years

Ref.	Heat source type	Heat source area	Heat flux (W/cm^2^)	Maximum temperature (°C)	COP	Work
^18^	Cartridge heater- based source	10 (2 × 5) mm^2^	1000–1200 (100–120 W)	120	197–236	Experimental
	GaN-on-SiC heat source dies (dummy)	6 (1.2 × 5) mm^2^	3000–4000 (180–240 W)		355–473	
^20–22^	GaN HEMT MMIC	–	HEMT: 31 k–38 k	182–212	–	Numerical
			Die: 1–1.25 k			
^23–25^	5 TaN resistors and 4 HEMTs	HEMT ~0.005 mm^2^	HEMT device: 30 k (1.6 W) Die Level: 1 k (131 W)	158 (Exp.) 135 (Num.)	–	Experimental and numerical
	HPA MMIC (substrate etching)	12.5 (2.5 × 5.0) mm^2^		161	345	Experimental
^27^	GaN-on-Si heat source	4.4 mm^2^	1.7 k (75 W)	85	2982	Experimental
	Schottky diode based full-wave bridge rectifier		417		1000	
This work	GaN HEMT device	Gate finger 0.018 (10 × 0.0018) mm^2^	32.8 k	93.8	3725	Experimental and numerical
		Active area 0.119 mm^2^	5 k			

## Conclusions

In conclusion, we presented a 2.5D integration method with chip-level direct microchannel cooling for a high-power GaN HEMT device. To demonstrate this technology, a GaN HEMT device with in-house backside thinning and metallization was used as a test vehicle, and an HR Si interposer embedded with four-layer microchannels was designed, with a top microchannel 30 μm/30 μm in width/pitch. A key process was developed to realize Si–Si wafer-level bonds with vias and microchannels on the bond surface. The GaN HEMT device was soldered onto the HR Si interposer above open microchannels, and a pair of GSG Pad chips was soldered in the same manner to showcase the capacity of integrating other kinds of chips. Thermal property evaluation was conducted in DC mode, the relationships of *I*_ds_ and the maximum surface temperature versus *V*_ds_ and DI water flow rate were investigated with simulations and experiments. A good agreement between the simulation and experimental results was obtained. When *V*_ds_ increased to 7.5 V, *I*_ds_ was measured to be approximately 0.8 A, and the maximum surface temperature was measured to be approximately 93.8 °C. In this case, the heat flux was 32,806 W/cm^2^ in the gate finger and 5044 W/cm^2^ in the active area. This finding was sufficient to verify the cooling capacity of the proposed technology. Since the junction temperature of the GaN HEMT device was far below the limit, there was still room for improvement. To our knowledge, among recently reported works, the result of this study displayed the best cooling capacity of heterogeneously integrated microchannels for GaN HEMT devices. In addition, the processes of the GaN HEMT device and HR Si interposer could be implemented separately, significantly improving the process compatibility. Since the straight parallel microchannels were aligned parallel with the gate fingers of the GaN HEMT device, the number of parallel microchannels could be increased according to the numbers of GaN HEMT device gate fingers in the applications. In this scenario, the device-level direct microchannel cooling capacity was scalable.

## Supplementary information

Supplemental Material

## Data Availability

The data that support the findings of this study are available within the article.
