# Supplementary material for: Thermal property evaluation of a 2.5D integration method with device level microchannel direct cooling for a high-power GaN HEMT device
Source: Microsyst Nanoeng. 2022 Nov 11;8:119. doi: 10.1038/s41378-022-00462-3 (PMC9649738; doi:10.1038/s41378-022-00462-3)
Supplement: Supplementary file 1 — Supplemental Material [file 41378_2022_462_MOESM1_ESM.docx]

### SUPPLEMENTAL INFORMATION

**Thermal property evaluation of a 2.5D integration method with device level microchannel direct cooling for a high-power GaN HEMT device**

Tingting Lian,^1^Yanming Xia,^1^ Zhizheng Wang,^1^ Xiaofeng Yang,^2^ Zhiwei Fu,^2^ Xin Kong,^3^ Shuxun lin,^4^ and Shenglin Ma^1,*^

**AFFILIATIONS**

^1^Department of Mechanical and Electrical Engineering, Xiamen University, Xiamen 361005, China

^2^China Electronic Product Reliability and Environmental Testing Research Institute, Guangzhou 510000, China

^3^ The 29th Research Institute of China Electronics Technology Group Corporation, Chengdu 610000, China

^4^ChengDu Gastone Technology CO., LTD., Chengdu 610000, China

1. Author to whom correspondence should be addressed: [mashenglin@xmu.edu.cn](mailto:mashenglin@xmu.edu.cn)

The design layout of the commercial GaN HEMT device used in this study is shown in Figure S-1. The size of the chip is 1120 μm (height)×878.5 μm (width)×100 μm (thickness), the active area is 303 μm×393 μm, the total area of the gate fingers is 10×300×6 μm^2^. The signal of source (S) is realized through the via for ground, as shown in Figure S-1(b) and (c).

| 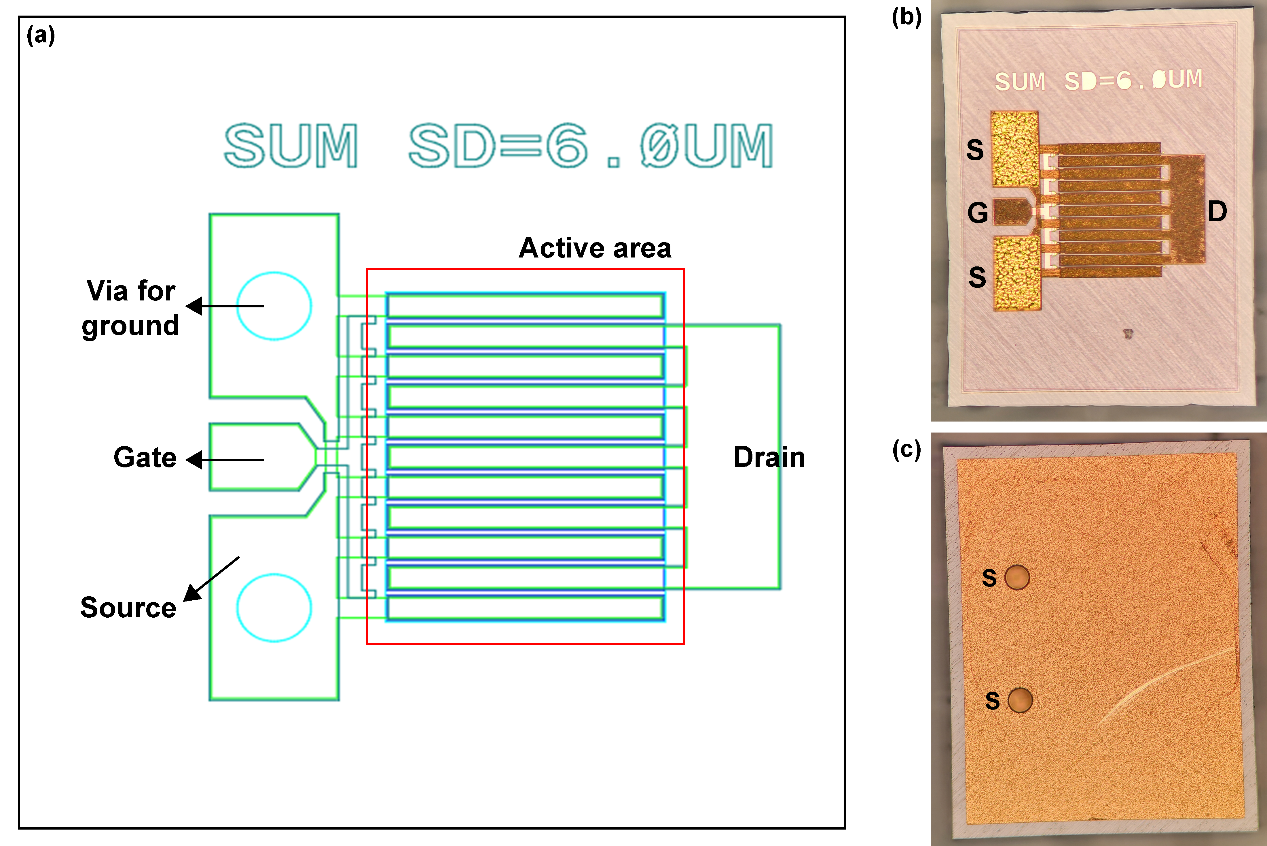  **Figure S-1.** The commercial GaN HEMT device used in this study. (a) The design layout of the GaN HEMT device. (b) Topdown view of the GaN HEMT device. (c) Bottom view of the GaN HEMT device. |
| --- |

AuSn eutectic bonding process is performed to bond the GaN chip to the corresponding position on the upper surface of the interposer. To evaluate the quality of bonding, corresponding tests were conducted. Firstly, the porosity test was conducted. As shown in Figure 2(a), the sample was loaded on the X-ray test platform (YXLON Ycheetah). Then the test result shows that the bonding porosity is 6.5%, as shown in Figure 2(b). Secondly, the warpage test was conducted. As shown in Figure 3(a), the sample was loaded on the bench test platform (CYBER technologies Cyber CT100). The result shows that the maximum warpage is 540 nm as shown in Figure 3(b). Finally, the shear strength test was conducted, and the sample was loaded on the shear strength test platform (XYZ TEC CONDOE-70), as shown in Figure 4(a). The test result shows that the shear strength is 4.860 kgf, as shown in Figure 4(b). As the bonding area is approximately 0.7451 mm^2^, the shear strength of the sample is calculated to be 63.921 Mpa. The above experimental results shows a quality within the acceptable range.


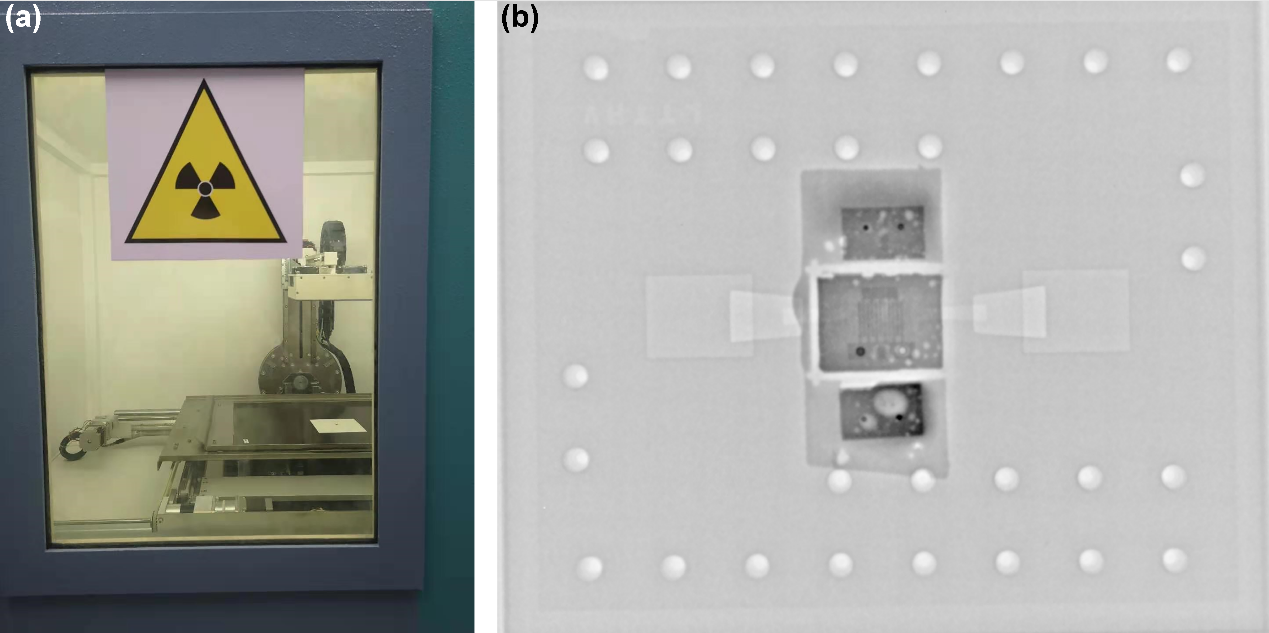


**Figure S-2.** X-ray to evaluate the porosity of AuSn solder. (a) X-ray test platform. (b) X-Ray image of the sample after solder bonding with the GaN HEMT device.


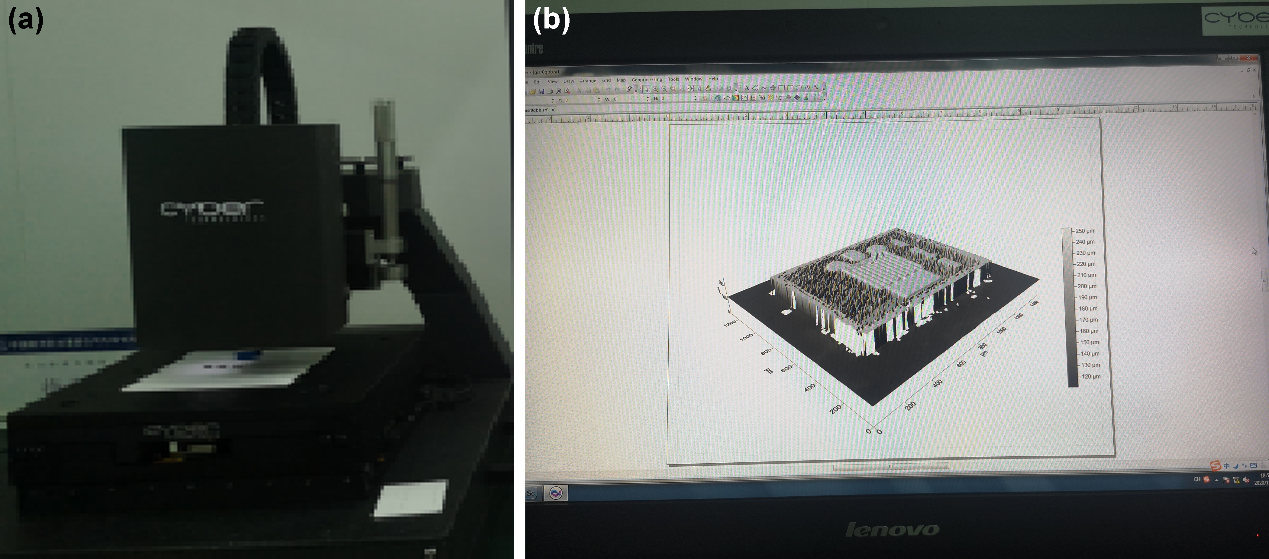


**Figure S-3.** Warpage test to evaluate the quality of sample after AuSn eutectic bonding. (a) Warpage test platform.(b) Warpage test interface.


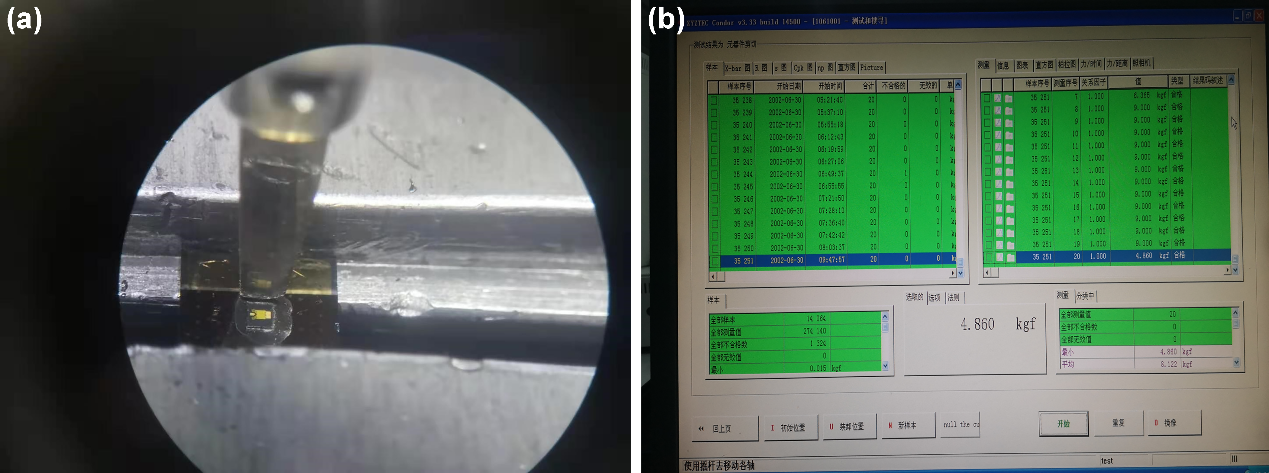


**Figure S-4.** Shear strength test to evaluate the quality of bonding. (a) The sample loaded on the shear stength test platform. (b) Shear strength test interface.

Figure S-5 shows the assembled sample for heat dissipation characteristic test. A pair of the GSG Pads were aligned and soldered to both sides of the GaN HEMT device simultaneously, as shown in Figure S-5(b). The topdown and bottom views of the GSG Pad are shown in Figures (c) and (d) respectively. Subsequently, the Si interposer was assembled to a customized aluminum alloy box and the gaps around inlet and outlet regions were sealed by DOWSIL™ 3145 adhesive. Gold wire bonding was used to interconnected the GaN HEMT device and the GSG Pads to fan out electrical signal, as shown in Figure S-5 (b).


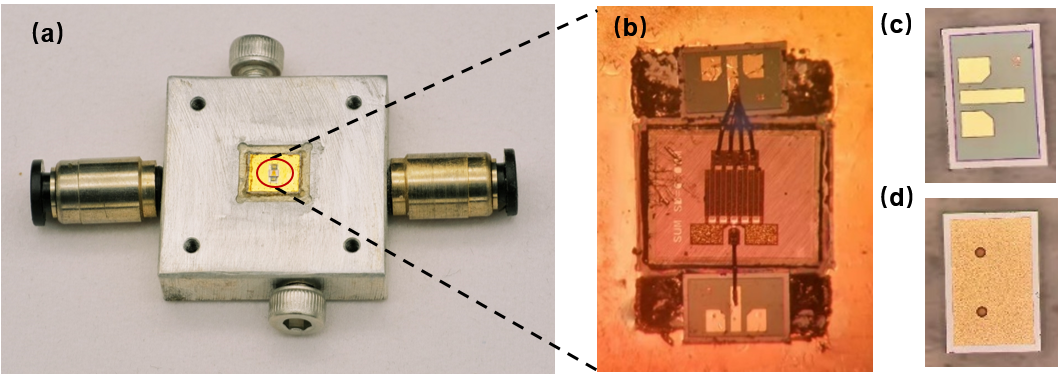


**Figure S-5.** The sample after assembly and gold wire bonding, ready for heat dissipation characteristic test. (a) The sample test cube. (b) The GSG Pads and the GaN HEMT device are bonded to the corresponding position on the surface of the interposer, and the gold wire bonding realizes the signal interconnection. (c) Topdown view of the GSG Pad. (c) Bottom view of the GSG Pad.

The experimental test platform to characterize the heat dissipation performance of the sample is shown in Figure S-6. A probe with a diameter of 80 μm was connected to the corresponding position of the GSG Pad, and then the cable of probe was connected to a DC stabilized source (ITECH IT6332B). A QFI infrared thermal imager (Figure S-6(b)) with a resolution of 2 μm is used to obtain the surface temperature contour of the GaN HEMT device.


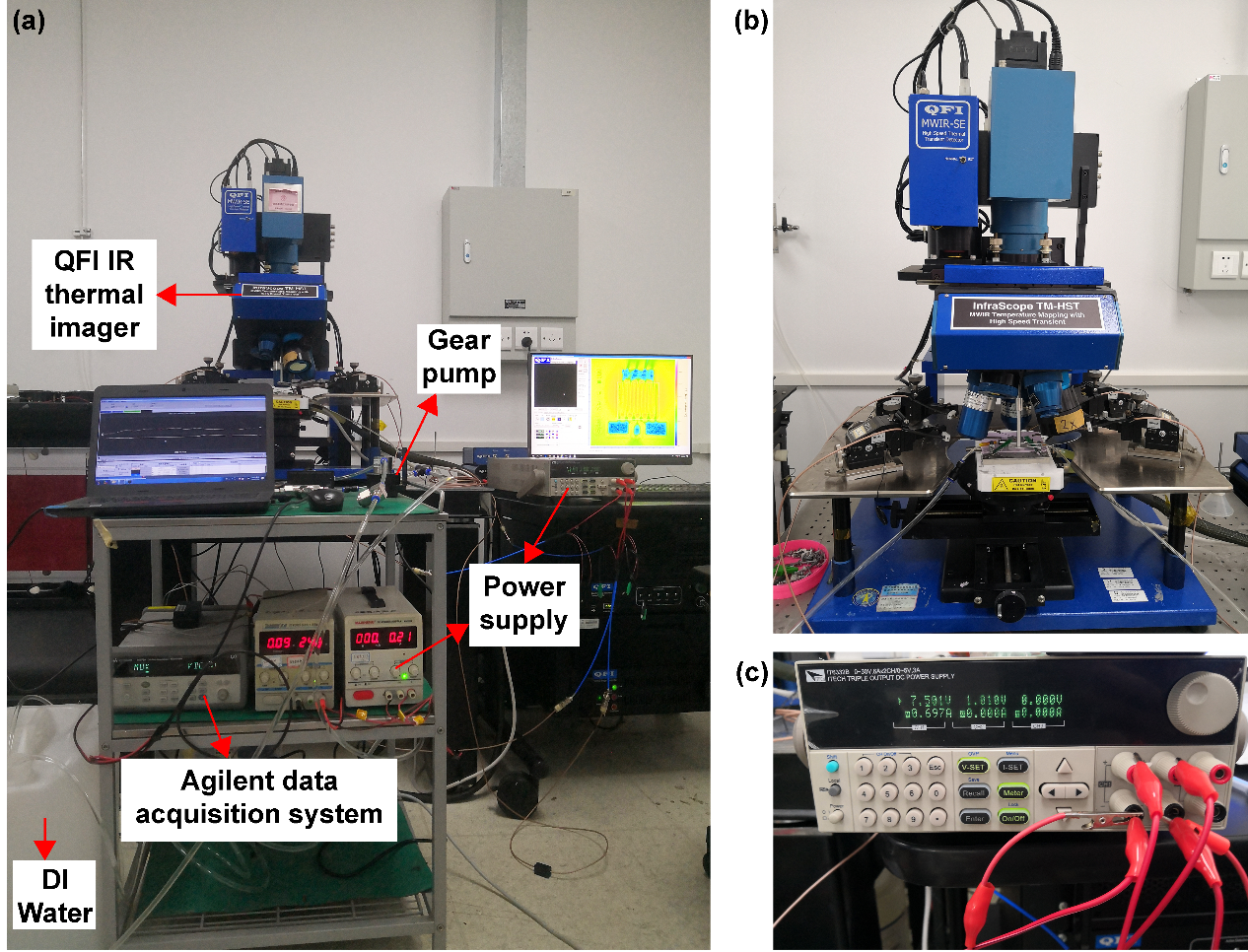


**Figure S-6.** Experimental test to characterize the heat dissipation performance of the sample. (a) Thermal test platform. (b) QFI infrared thermal imager. (c) DC power supply.
